# Recycling Nutrient Solution Can Reduce Growth Due to Nutrient Deficiencies in Hydroponic Production

**DOI:** 10.3389/fpls.2020.607643

**Published:** 2020-12-22

**Authors:** Alexander Miller, Ranjeeta Adhikari, Krishna Nemali

**Affiliations:** Department of Horticulture and Landscape Architecture, Purdue University, West Lafayette, IN, United States

**Keywords:** fertilizer solution, macronutrients, micronutrients, segmentation, water quality

## Abstract

It is common in hydroponics to supply nutrients to crops by maintaining electrical conductivity (EC) of the recycling solution at a target level. Levels of individual nutrients in the solution are generally not assessed as their regular measurement and adjustment can be both expensive and technically challenging. However, the approach of growing crops at a target EC can potentially result in nutrient imbalances in the solution and reduced growth. We quantified the effects of recycling on solution EC changes, tissue nutrient concentration, canopy growth rate, plant water status, and shoot and root weight of lettuce (*Lactuca sativa*) in a greenhouse. The tap water quality was moderately alkaline and similar to that commonly observed in many commercial greenhouses. In our research, recycling solution maintained at a target EC (1.8 dS⋅m^–1^) significantly reduced shoot fresh (22–36%) and dry weight compared to the control supplied regularly with freshly prepared solution at the target EC. Further, recycling significantly decreased N, P, K, and Fe and increased Na and Cu levels in the tissue, in addition to increasing solution EC between adjustments compared to the control. Using image analysis of groups of plants, we identified that the negative effects of recycling on canopy area started 2 weeks after transplanting. Based on these results, we hypothesized that certain unwanted compounds (e.g., bicarbonates) and slowly consumed elements (e.g., Ca, Mg) were added to the recycling solution through the alkaline tap water with time. Their accumulation “artificially” increased solution EC and “masked” the lower than optimal levels of major nutrients in the solution, leading to the reductions in the concentration of nutrients in the tissue and plant growth. Supporting this, the negative effects of recycling were not observed when the recycling solution was either discarded after 2 weeks of use or made using reverse osmosis water and continuously used. Our findings aid in proper management of recycling solution in hydroponic lettuce production.

## Introduction

Hydroponics industry is becoming popular in the United States with nearly 2500 enterprises and overall revenue of $ 0.83 billion in 2019 (Us Specialized industry report, 2019). The increased demand for fresh, locally grown, and safe food is driving the growth of the industry in the United States Lettuce accounts for nearly 7% of overall share of the industry in the United States (Us Specialized industry report, 2019). Hydroponic lettuce is mostly grown in a solution enriched with nutrients and oxygen using different production systems including nutrient film, deep flow, floating bed, and ebb and flow systems (Resh, 2012; Son et al., 2020).

Nutrient solution is usually recycled during hydroponic production (Jensen, 1997; Nederhoff and Stanghellini, 2010) to reduce wastage. Electrical conductivity (EC) of the recycling solution is measured to determine its nutrient status (Brun et al., 2000; Christie, 2014; Jones, 2016). The EC measurement is an indirect and quick-way to measure the total concentration of ions, including nutrients, dissolved in a solution (Graves, 1983; Nemali, 2018). A common practice in hydroponics is to maintain a target EC level in the recycling solution by frequently measuring and adjusting EC using water and nutrients. It is assumed that adequate amount of nutrients can be made available to plants by maintaining the recycling solution EC at the target level. However, maintaining solution EC at a target level may not necessarily result in optimal concentration of individual plant nutrients in the recycling solution. It is not possible to assess the levels of individual ions dissolved in the solution based on EC measurements (Nemali, 2018). Without knowing the levels of individual plant nutrients, it is difficult to ensure their optimal levels in the solution. Therefore, despite maintaining solution EC at a target level, it is possible that certain plant nutrients can become excess or deficient in the recycling solution with time.

Good quality irrigation water can be a limiting factor in many commercial hydroponic greenhouses (Allende and Monaghan, 2015). Irrigation water quality is alkaline in many parts of the United States Midwest (Kaushal et al., 2018), due to the presence of bicarbonates (HCO_3_) of calcium (Ca) and magnesium (Mg) (Kaushal et al., 2013, 2018; Guo et al., 2015). When irrigation water with high alkalinity is used in production, Ca, Mg, and HCO_3_ can accumulate in the recycling solution (Baars, 1992; Carmassi et al., 2003; Trejo-Téllez and Gómez-Merino, 2012; Sambo et al., 2019). This is because HCO_3_ can’t be transported through the plant roots (Poschenrieder et al., 2018) while Ca and Mg are slowly consumed by plants (Bugbee, 2004). A direct effect of their accumulation is an “apparent” increase in the recycling solution EC (Zekki et al., 1996). It is possible for solution EC to be close to target EC even when the concentration of major plant nutrients is sub-optimal, due to increased concentration of Ca, Mg, and HCO_3_ in the recycling solution. In addition, sodium (Na) in irrigation water can accumulate in the recycling solution (Fernandez, 2017) and cause osmotic stress to plants (Hopkins et al., 2007). There is limited research that quantified the negative effects of using recycling nutrient solution during production and identified physiological reasons for the observed effects of recycling on plant growth in hydroponics.

In addition, research that aimed at developing practical remedies to minimize recycling effects on plant growth is minimal. In some large commercial operations, recycling solution is regularly analyzed in a laboratory and the levels of individual nutrients are adjusted using complicated worksheets on computers. In addition to being expensive, adjusting individual nutrients regularly can be technically challenging to growers. Thus, many growers prefer to discard the recycling solution (Zekki et al., 1996; Lykas et al., 2006; Samarakoon et al., 2006) instead of managing the concentration of individual plant nutrients. However, there are no established guidelines on when to discard the recycling solution. Regardless of species and growth environment, the recycled solution should be discard when the negative effects on plant growth just start to appear. This approach can minimize fertilizer wastage and reduce environmental pollution resulting from frequent discarding. For this, continuous plant growth monitoring is needed to determine the correct stage for discarding. Shoot growth of leafy greens can be monitored by destructively harvesting and weighing plants (Li et al., 2020). However, regular destructive harvests may not be popular due to plant loss. In addition, regular destructive harvests increase labor costs. Currently, there are limited choices for non-destructive crop growth assessments in hydroponic production. Image analysis can be used to non-destructively assess lettuce growth (Li et al., 2020). It is also possible to use image-based measurements for continuous plant growth monitoring on easy-to-use devices like smartphones (Li et al., 2020). However, the efficacy of image analysis technique for timely detection of the negative effects of recycling on plant growth was never tested in hydroponic production.

The aims of the study were to evaluate the effects of continuous recycling on solution EC, tissue nutrient concentration and productivity of lettuce, and develop optimal strategies for managing recycling nutrient solution in hydroponic production. Specifically, our objectives were to (i) quantify the effects of recycling solution on lettuce (*Lactuca sativa*) growth, (ii) identify the stage when recycling effects are observed on plants using image analysis, (iii) relate observed effects of recycling on plant growth to measured physiological responses, and (iv) develop practical remedies to minimize the effects of recycling on lettuce growth.

## Materials and Methods

### Plant Materials and Growth Conditions

We conducted three separate experiments in the study. Experiment 1 was intended to quantify the effects of recycling on plant growth. We designed experiment 2 to understand physiological reasons for the observed effects of recycling in experiment 1 and identify the stage when recycling effects are observed on plants using image-based assessments. Experiment 3 was conducted to validate a hypothesis developed in experiment 2 and identify remedies for minimizing recycling effects in commercial production. We grew leaf lettuce (cv. Black Seeded Simpson) in experiments 1 and 2 because of its fast growth rate, therefore increased probability to detect growth differences. In experiment 3, we used cultivars of lettuce belonging romaine (cv. Amadeus), leaf (cv. Black Seeded Simpson), butterhead (cv. Rex), and oakleaf (cv. Cedar) groups.

Plant materials, seedling production, and growth environment were similar in all experiments. Seeds (Paramount Seeds Co., Stuart, FL, United States) were sown in sheets of rock wool cubes (200 per sheet, 2.5 cm diameter each, Grodan, Roermond, Netherlands) that were placed on watertight trays (54 cm × 27 cm × 3 cm; Greenhouse Megastore, Danville, IL, United States) for sub-irrigation. The trays were filled every day with approximately one liter of tap water to keep the rock wool cubes moist. After emergence, we thinned the seedlings to one per cube and sub-irrigated them with a dilute nutrient solution containing nitrogen at a concentration of 50 mg⋅L^–1^. A water-soluble fertilizer containing 20 N-4.4 P-16.6 K (20-10-20, The Scotts Co., Marysville, OH, United States) mixed with tap water was used to prepare the nutrient solution. Seedlings were transplanted into hydroponic production systems (see “Hydroponic Systems” section below) after 10 days from the sowing. Plants were grown in a glass greenhouse located at Purdue University, West Lafayette, IN, United States. The daily average (standard deviation) air temperature, light integral, and relative humidity in the greenhouse during the experiments were 23.7 ± 1.81°C, 10.5 ± 3.82 mol⋅m^–2^, and 80.2 ± 8.76%, respectively.

### Hydroponic Systems

**FIGURE 1:**
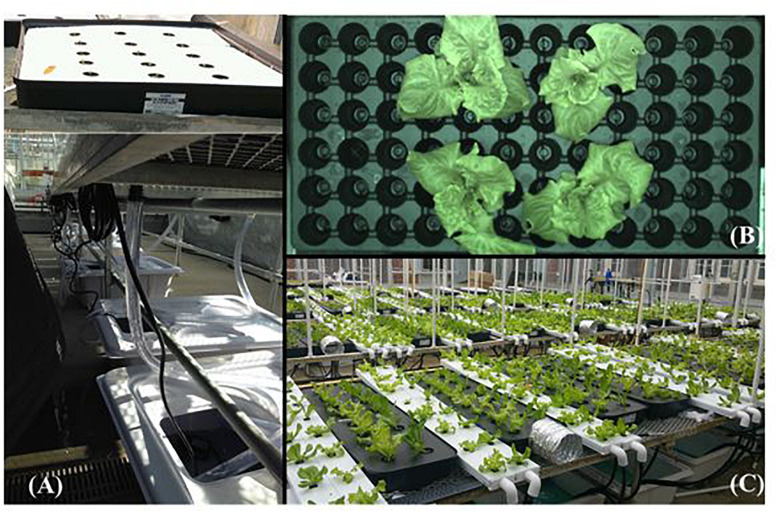
Custom-built hydroponic production systems used in experiments 1 **(A)**, 2 **(B)**, and 3 **(C)**. See “Materials and Methods” section for a description about production systems.

A custom-built hydroponic production system similar to commercial flood tables was used in experiment 1 (Figure 1A). It was built using black plastic trays (91 cm × 91 cm × 10 cm, 82.8 L volume; Botanicare, Vancouver, WA, United States), nutrient solution reservoirs (76 L; Active Aqua Premium White Reservoir, Petaluma, CA, United States), submersible pumps (9.5 L min^–1^; TotalPond, West Palm Beach, FL, United States), and vinyl tubing (0.013 m internal diameter; CropKing Inc., Lodi, OH, United States). The trays with covered lids (1.3 cm Styrofoam, U-Line, Pleasant Prairie, WI, United States) were arranged on a greenhouse bench (7.6 m × 1.5 m × 1.1 m). The lids contained holes for inserting net pots (5.1 cm diameter; General Hydroponics, Chico, CA, United States). Reservoirs stored approximately 20 L of the nutrient solution, which was continuously recycled during production. An extension fitting was inserted in the outlet end to enable the nutrient solution to accumulate in the tray before draining back to the reservoirs. The depth to which nutrient solution accumulated in the tray was approximately 1.0 cm. At steady state, approximately 8 and 12 L of recycled solution was present in the flood tables and reservoirs, respectively. Each tray housed 15 net pots in five rows of three each. Each net pot contained one rock wool cube with a germinated seedling. The net pots were spaced 23 cm apart within a row and 15 cm apart between the rows. The base of the net pot rested on the bottom of the tray after inserting through the hole, thereby exposing the lower portion of rock wool cube to the nutrient solution.

Experiment 2 required frequent plant movement and measurements of solution volume (see “Measurements” section below). To facilitate this, we grew plants in hydroponic seeding inserts (72 cell, Hydrofarm, Greenhouse Megastore, Danville, IL, United States; Figure 1B) placed on watertight trays (9.4 L; 1020 tray; 54.5 cm × 27.8 cm × 6.2 cm, Greenhouse Megastore). The trays were arranged on the greenhouse bench. Each tray stored 2 L of nutrient solution to a depth of approximately 1.5 cm. Although plants were grown in a “passive” hydroponic system, a significant volume of nutrient solution was changed daily (see “Treatments” section below) to ensure that oxygen levels were not compromised. The rock wool cubes with seedlings were directly placed in the cells of the seeding inserts. In each tray, four rock wool cubes with seedlings were arranged in two rows of two each. The spacing between plants was 17 cm within a row and 13 cm between the rows. The bottom of the insert cells was cut open to ensure that the base of the rock wool cubes were in direct contact with the nutrient solution.

Plants in experiment 3 were grown in custom-built constant flood table (CFT) and nutrient film technique (NFT) systems (Figure 1C). We built the CFT and NFT systems using reservoirs and submersible pumps similar to those described for experiment I. In addition, black flood tables (122 cm × 31 cm × 10 cm; Botanicare, Vancouver, WA, United States) and lids for the CFT and white channels (150 cm × 12 cm × 4 cm; CropKing Inc., Lodi, OH, United States) and covers for the NFT were purchased from the respective vendors to build the hydroponic systems. The reservoirs stored approximately 20 L of nutrient solution. Black vinyl tubing (1.3 cm internal diameter; Crop King Inc.) was used to connect the pumps to the two production systems and drain the nutrient solution back to the reservoirs. To allow for uniform flow from the inlet to outlet end, the NFT channels were raised by 0.15 m (9.8% slope). An extension fitting (5 cm; Botanicare) was inserted into the outlet of the CFT tray for solution to accumulate (approximately 4 cm depth) in the tray before draining. Flow valves (1.3 cm; Green Back in-line valve, Botanicare) connected to the inlet tubing were used to control the rate of nutrient solution delivery to the CFT and NFT systems. The measured flowrate on the outlet end of the CFT and NFT systems was approximately 6.0 and 1.0 L⋅min^–1^, respectively. Two NFT channels (one unit) and one CFT tray (one unit) were connected to a reservoir. Each production system unit housed 16 plants belonging to four cultivars with four plants each. The rock wool cubes with seedlings were transplanted in the NFT system by inserting through the square holes (3 cm) on the channel covers and making them contact with the base and solution flowing through the channel. The holes were spaced 18 cm within a channel and 13 cm between the channels. In the CFT system, rock wool cubes with seedlings were placed in net pots (General Hydroponics), which were inserted into the holes (6 cm) made on the CFT lids. During the steady flow state, nearly three-fourth of the rock wool cube was immersed in the nutrient solution. The holes on the CFT lids were spaced 17 cm apart within a row and 13 cm apart between the rows.

### Treatments

Plants were exposed to two solution treatments (“recycle” and “control”) in experiments 1 and 2. Nutrient solution was prepared by mixing the same water-soluble fertilizer used during the seedling stage in both experiments. The target nutrient solution EC in both the treatments was 1.8 dS⋅m^–1^ (pH between 6.2 and 7.1). The EC measurement included the contribution of dissolved salts in the tap water (approximately 0.7 dS⋅m^–1^). In the control treatment, old solution in the trays and reservoirs was discarded and either 20 (experiment 1) or 2 L (experiment 2) of freshly prepared nutrient solution with an EC of 1.8 dS⋅m^–1^ (corresponding to 166 mg N⋅L^–1^) was added to the reservoirs three times a week (experiment 1) or daily (experiment 2). In the recycle treatment, the leftover nutrient solution in the trays and reservoirs was retained. However, solution volume (20 L in experiment 1 and 2 L in experiment 2) and target EC (1.8 dS⋅m^–1^) were maintained, either three times a week (experiment 1) or daily (experiment 2). For this, the volume of leftover solution in the reservoirs (experiment 1) and trays (experiment 2) was measured and adjusted to the target volume (20 or 2 L, respectively) using the tap water. The solution EC was measured after adjusting for the volume by adding a fertilizer stock solution to increase the solution EC to the target level of 1.8 dS⋅m^–1^.

In experiment 3, plants were exposed to three solution treatments [control, 2 week discard (or 2 WkD), and recycling with reverse osmosis (RO) water (or Rec_RO)], two production system (CFT and NFT) treatments, and four cultivar treatments (Amadeus, Black Seeded Simpson, Cedar, and Rex). The control treatment was similar to that described above for experiments 1 and 2. The 2 WkD treatment was similar to the recycle treatment described above for experiment 1, except that the old nutrient solution was discarded 2 weeks after use and reservoirs were refilled with 20 L of freshly prepared solution at target EC. Recycling continued with periodic EC adjustment to target level. The Rec_RO treatment was similar to recycle treatment described above for experiment 1, except that RO water was used instead of tap water to grow plants. The target EC of Rec_RO treatment was 1.1 dS⋅m^–1^, a value equivalent to the EC solely due to dissolved fertilizer ions in other treatments. The EC of the solution was regularly adjusted to the target EC as described above for experiment 1. The differences between the two production systems were previously described in the “Hydroponics Systems” section. The cultivars belonging to different groups varied in their growth rates.

### Measurements

Light intensity was measured using quantum sensors (SQ110, Apogee Instruments, Logan, UT, United States) placed at different locations on the bench. Air temperature was measured using aspirated temperature sensors (ST 110, Apogee Instruments) placed in the proximity of the quantum sensors. Both sensors were connected to a datalogger (CR1000X, Campbell Scientific, Logan, UT, United States) for continuous measurements. Daily light integral and daily average temperature were calculated from the instantaneous light intensity and temperature measurements, respectively. Additional temperature sensors were connected to the datalogger in experiment 3 to measure solution temperature in the CFT and NFT systems. Relative humidity measurements were obtained from the environmental control system (Priva, Canada) in the greenhouse. The EC and pH of the nutrient solution were measured using pH/TDS/EC meter (Model #HI9811, Hanna Instruments, Ann Arbor, MI, United States).

In experiment 1, we measured shoot fresh weight (SFW), shoot dry weight (SDW), and root dry weight (RDW) of plants. Plants were harvested on the 22nd day after transplanting. Shoots and roots from a flood table were separated from the rock wool cubes and SFW of all plants was recorded along with the number of plants. From this data, SFW per plant (g⋅plant^–1^) was calculated by dividing total fresh weight of all plants by the number of plants in a tray. The shoot and root materials from each tray were placed in separate paper bags and dried in a forced air oven maintained at 80°C for 1 week. The dried material was weighed to determine SDW and RDW. From these, SDW and RDW per plant (g⋅plant^–1^) were calculated by dividing the total dry weight by the number of plants. Root weight ratio (RWR) (dimensionless) was calculated by dividing RDW by the sum of RDW and SDW. Shoot water content (SWC), (%) was calculated by dividing water weight (SFW minus SDW) by SFW and multiplying the result by 100.

In experiment 2, we measured canopy area (CA, cm^2^), relative canopy growth rate (RCGR) (d^–1^), EC of the nutrient solution prior to adjustment (EC_adj_, dS⋅m^–1^), end-of-day solution volume (V, L), SFW, SDW, and concentration of different nutrients in the tissue. We used an imaging station (TopView, Aris, Eindhoven, Netherlands) to non-destructively measure CA of plants on different days. Whole-trays with plants were placed inside the image station on each measurement day for capturing images. The height of the tray from the base of the image station was adjusted to ensure that the distance between the camera and top of the plants was similar during each measurement. The images of shoots were captured as the roots were completely covered by the insert. The image processing software automatically separated plants from the background and calculated total pixel area belonging to the plants in each image. The CA (mm^2^) was automatically estimated by multiplying the total plant pixel area with a constant (or “magnification factor” of 100) specific to the image station and converting to cm^2^. We measured CA on the 10th, 13th, 15th, 18th, 20th, 21st, and 22nd day of the experiment. RCGR was measured as the slope of the linear relationship between ln (CA) and time. Left over solution in each tray was collected into a beaker to measure V around 4.00 pm each day. The evapotranspiration rate (ET, L⋅d^–1^) was calculated by subtracting V from 2 L. The EC of the solution in the beakers was used to measure EC_*adj*_. Plants were harvested on the 22nd day after transplanting. Both SFW (g⋅plant^–1^) and SDW (g⋅plant^–1^) were measured as described above. The dried shoot material was grinded, and a representative sample was extracted from each tray. The samples were sent to a commercial laboratory (A&L Great Lakes, Fort Wayne, IN, United States) for complete elemental analysis.

In experiment 3, we measured SFW (g⋅plant^–1^), SDW (g⋅plant^–1^), RDW (g⋅plant^–1^), and EC_adj_ (dS⋅m^–1^) as described above. We measured the temperature of the solution inside three randomly selected CFT and NFT systems. The measurements of solution temperature were made continuously for seven consecutive days prior to the harvest. From this, hourly average temperature and standard deviation were calculated. In addition, samples of tap water and RO water used in the experiments were sent to the same commercial laboratory described above for water quality analyses.

### Experimental Design and Statistical Analyses

Experiment 1 was laid-out in a randomized complete block design with two treatments and six replications. In each replication, solution treatments were represented by separate reservoirs. An experimental unit comprised of fifteen plants on a flood table belonging to a solution treatment and replication. Experiment 2 was also laid-out in a randomized complete block design with two treatments and nine replications. An experimental unit comprised of four plants in a tray belonging to a solution treatment and replication. Experiment 3 was laid-out in a split-plot design with seven replications of main-plot. The solution treatment was as the main-plot, production system was the sub-plot, and cultivar was the second sub-plot. A reservoir belonging to a solution treatment in a replication was connected to one CFT and one NFT unit. There were four plants each of four cultivars in one production system unit. An experimental unit comprised of four plants belonging to a cultivar within a production system and solution treatment in a replication. In all experiments, the treatments were randomly allotted to experimental units. Data were analyzed using a linear-mixed model (“Proc Mixed” procedure) with repeated measures and linear/non-linear regression (Proc “Reg” and Proc “Nlin”) using statistical analysis software (SAS, version 9.1, SAS Institute, Cary, NC, United States). Least square means were separated using Tukey’s honestly significant difference (HSD) procedure with *P* ≤ 0.05 considered statistically significant. Graphs were plotted using SigmaPlot (version 14, Systat Software Inc., San Jose, CA, United States).

## Results

### Experiment 1

A significant reduction in SFW of lettuce was observed in the recycle compared to control treatment (Table 1). SDW of lettuce in the recycle treatment was numerically lower than that of control treatment. However, there were no differences in RDW and RWR between the recycle and control treatments. SWC was significantly higher in the control compared to recycle treatment.

**TABLE 1:** Shoot fresh weight (SFW), shoot dry weight (SDW), root dry weight (RDW), root weight ratio (RWR), and shoot water content (SWC) of leaf lettuce in experiment 1.

Treatment	SFW	SDW	RDW	RWR	SWC
			
	g⋅plant^–1^	g⋅plant^–1^	g⋅plant^–1^		%
Control	31.0 (4.35) a	3.5 (0.61) a	3.0 (0.84) a	0.42 (0.038) a	88.8 (1.01) a
Recycle	20.9 (3.61) b	3.3 (0.57) a	2.9 (0.78) a	0.43 (0.033) a	83.9 (1.40) b

*Means followed by the same letter are not statistically different (*P* ≤ 0.05). Standard error of mean is shown in parenthesis.*

### Experiment 2

Similar to experiment 1, a significantly lower SFW was observed in the recycle compared to control treatment (Table 2). Decrease in SDW of lettuce was small but significantly lower in the recycle compared to the control treatment. When EC_*adj*_ was compared, a significant increase was observed in the recycle compared to control treatment. However, there were no significant differences in ET between the recycle and control treatments. Concentration of several nutrients in the tissue including nitrogen (N), phosphorus (P), potassium (K), and iron (Fe), were significantly lower in the recycle compared to the control treatment (Table 3). Tissue analysis indicated significantly higher levels of copper (Cu) and sodium (Na) in the recycle compared to control treatment.

**TABLE 2:** Shoot fresh weight (SFW), shoot dry weight (SDW), electrical conductivity of nutrient solution before adjustment to the target level (EC_*adj*_), evapotranspiration rate (ET), and relative canopy growth rate (RCGR) of leaf lettuce in experiment 2.

Treatment	SFW	SDW	EC_*adj*_	ET	RCGR
			
	g⋅plant^–1^	g⋅plant^–1^	dS⋅m^–1^	L⋅d^–1^	d^–1^
Control	35.3 (0.89) a	1.3 (0.05) a	2.3 (0.05) b	0.73 (0.041) a	0.191 (0.0064) a
Recycle	27.6 (0.61) b	1.2 (0.05) b	2.6 (0.07) a	0.74 (0.042) a	0.164 (0.0038) b

*Means followed by the same letter are not statistically different (*P* ≤ 0.05). Standard error of mean is shown in parenthesis.*

**TABLE 3:** Concentration of nutrients in the tissue of lettuce in experiment 2.

Nutrient	Units	Treatment	
		Control	Recycle
N	(mg⋅g^–1^)	37.6 (1.29) a	24.3 (0.70) b
P		5.4 (0.11) a	2.7 (0.20) b
K		42.0 (1.15) a	25.4 (1.01) b
Ca		10.7 (0.40)	11.0 (0.34)
Mg		5.2 (0.20)	5.3 (0.27)
S		3.2 (0.17)	3.0 (0.12)
Na		1.9 (0.08) b	3.2 (0.20) a
B	(mg⋅kg^–1^)	38.8 (2.37)	33.4 (1.79)
Zn		43.1 (2.72)	39.8 (3.16)
Mn		91.8 (5.24)	77.7 (8.65)
Fe		74.2 (8.41) a	46.3 (5.73) b
Cu		13.2 (1.56) b	20.0 (2.68) a
Al		47.4 (9.01)	53.6 (15.44)

*Mean values for nitrogen (N), phosphorus (P), potassium (K), calcium (Ca), magnesium (Mg), sulfur (S), sodium (Na), boron (B), zinc (Zn), manganese (Mn), iron (Fe), copper (Cu) and aluminum (Al) are shown. Means followed by the same letter within a measurement are not statistically different (*P* ≤ 0.05). Values in parenthesis indicate standard error of mean.*

**FIGURE 2:**
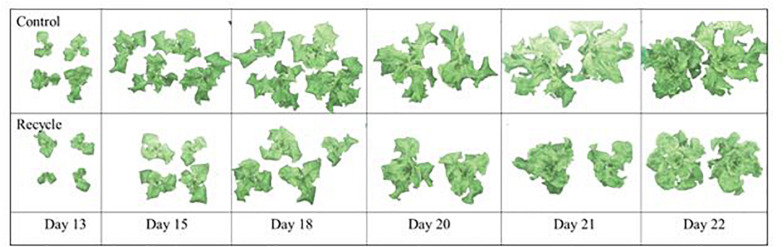
Images of leaf lettuce plants on different days showing segmentation (i.e., background removal) results in experiment 2. Plants were grown in the control and recycle solution treatments. See “Materials and Methods” section for a description of treatments. Images were captured using a TopView image station. Due to large plant size, images of two plants are included in the panels for days 20, 21, and 22. Note visual differences between the two solution treatments start to appear by day 13 and progressively become larger by day 22.

**FIGURE 3:**
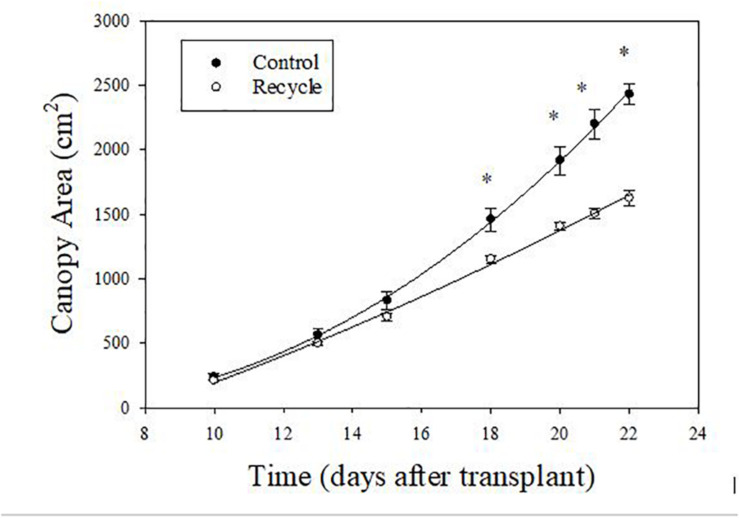
Change in canopy area of plants with time in experiment 2. Canopy area was non-destructively estimated using image analysis. Plants were grown in the control and recycle solution treatments. See “Materials and Methods” section for a description of treatments. Error bars represent standard error of mean. An asterisk (*) indicates statistical difference (*P* ≤ 0.05) between the means on a given day.

**FIGURE 4:**
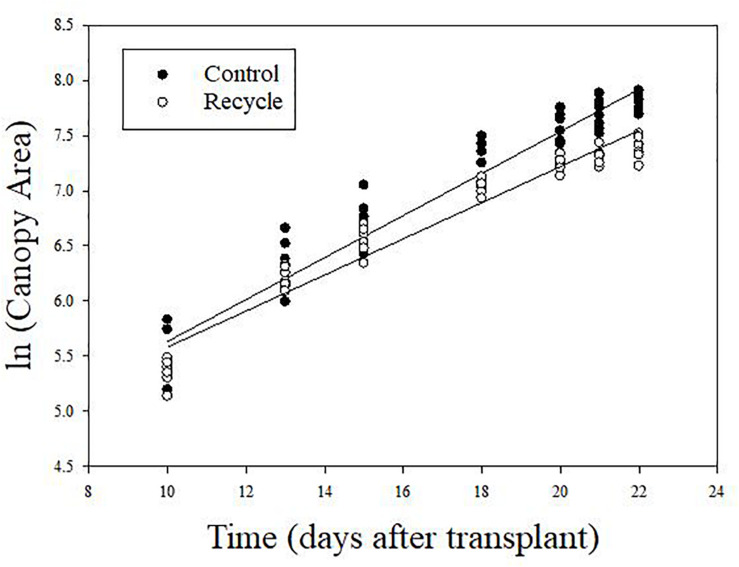
Relationship between natural logarithm of canopy area and time in experiment 2. Plants were grown in the control and recycle treatments. See “Materials and Methods” section for a description of treatments. The fitted equations are ln (CA_*control*_) = 3.73 + 0.191⋅time (*r*^2^ = 0.93) and ln (CA_*recycle*_) = 3.94 + 0.164⋅time (*r*^2^ = 0.94) for control and recycle treatments, respectively. The slope of the fitted equations represents relative canopy growth rate of plants.

The image analysis method effectively separated plant area from the background (Figure 2). Canopy area assessments indicated that the differences between the control and recycle treatments, although not significant, started earlier by the second week after transplanting. However, CA of plants in the control treatment was significantly higher than that of the recycle treatment starting from the 18th day after imposing treatments (Figures 2, 3). The smaller differences in canopy area became significantly larger with time. By day 22, canopy area of plants in the recycle treatment was approximately 33% smaller compared to that of plants in the control treatment (Figure 3). There was a linear relationship between ln (CA) and time in both the control and recycle treatments (Figure 4). The overall *r*^2^ for fitted models ranged between 0.93 and 0.94. RCGR of plants was significantly higher in the control than recycle treatment (Table 2, also see slope of the fitted models in Figure 4).

### Experiment 3

**FIGURE 5:**
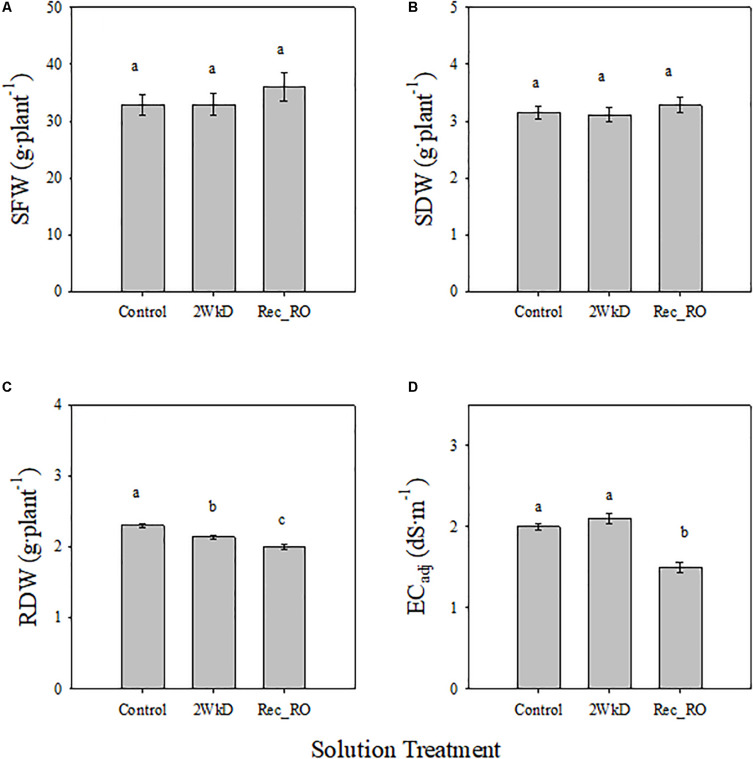
Effect of solution treatments on lettuce shoot fresh weight (SFW) **(A)**, shoot dry weight (SDW) **(B)**, root dry weight (RDW) **(C)**, and electrical conductivity of nutrient solution before adjustment to the target level (EC_*adj*_; **D**) in experiment 3. Plants in the control, 2 week discard (2 WkD) and recycling with reverse osmosis water (Rec_RO) treatments. See “Materials and Methods” section for a description of treatments. Different letters above bars indicate statistical significance (*P* ≤ 0.05) among treatments. Error bars represent standard error of mean.

There were no significant differences in SFW and SDW of lettuce among control, 2 WkD, and Rec_RO treatments (Figures 5A,B). However, significant differences were observed for RDW among treatments (Figure 5C). It was significantly higher in the control than 2 WkD and Rec_RO treatments. Further, RDW was significantly higher in the 2 WkD compared to the Rec_RO treatment. There were no differences in EC_adj_ between the control and 2 WkD treatments (Figure 5D). As expected, EC_adj_ was significantly lower in the Rec_RO compared to other treatments. Water quality analyses indicated higher EC and alkalinity in the tap water compared to RO water (Table 4). The concentration of Ca, Mg, S, Cl, Fe, Si, and HCO_3_ were higher in the tap water than RO water. There were no differences in pH between the tap water and RO water samples.

**TABLE 4:** Analysis of tap water and reverse osmosis (RO) water used in experiment 3.

Measurement	Units	Irrigation Water	RO Water
pH	n.a.	7.5	7.1
EC	(dS⋅m^−1^)	0.7	0.06
NO_3_-N	(mg⋅L^−1^)	0.8	–
NH_4_-N		– ^$^	–
P		1.1	–
K		3	–
Ca		102	–
Mg		38	–
Na		14	13
S		35	–
Zn		–	–
Mn		0.1	–
Fe		0.47	–
Cu		0.05	0.03
B		0.03	0.03
Al		–	–
Mo		–	–
Si		7	1
Cl		38	3
Alkalinity		250	20
CO_3_		–	–
HCO_3_		305	24

*Values for electrical conductivity (EC), nitrate-nitrogen (NO_3_-N), ammonium-nitrogen (NH_4_-N), phosphorus (P), potassium (K), calcium (Ca), magnesium (Mg), sodium (Na), sulfur (S), zinc (Zn), manganese (Mn), iron (Fe), copper (Cu), boron (B), aluminum (Al), molybdenum (Mo), silicon (Si), carbonate (CO_3_), and bicarbonate (HCO_3_) are shown.*

*^$^Undetected or levels are lower than the sensitivity of equipment.*

There was a significant effect of production system on lettuce growth. While SFW, SDW, and RDW were significantly higher, RWR was significantly lower in the NFT compared to CFT system (Supplementary Table 1). The difference between the solution and air temperature was consistently higher in the CFT than NFT system, especially during the daytime (Supplementary Figure 1). Among the four cultivars, SFW and SDW were significantly higher while RWR was significantly lower in Black Seeded Simpson than other cultivars (Supplementary Table 2). No differences were observed among Amadeus, Cedar, and Rex. In addition, there were no differences in RDW among all four cultivars.

## Discussion

### Effects of Recycling Nutrient Solution on Plant Growth

We compared plant responses in the recycle treatment with those in the control treatment because maximum plant growth is expected in the control treatment as freshly prepared nutrient solution at target EC was regularly supplied to plants. Therefore, the observed differences between the control and recycle treatment should reflect maximum loss in yield due to recycling nutrient solution. We observed significant reduction in SFW of lettuce due to recycling (Tables 1, 2) in spite of regularly maintaining the solution EC close to the target level in the recycle treatment. This indicates that maintaining target EC of the recycling solution does not necessarily result in optimal plant growth. Our results indicate that negative effects canopy area can start to appear as early as 2 weeks of recycling based on image analysis measurements (Figure 2). Decreased canopy area likely resulted in decreased SFW in the recycling treatment as canopy area can affect light interception and biomass production in plants (Niinemets, 2010; Li et al., 2020).

Tissue nutrient levels can potentially indicate reasons for the observed differences in canopy area and SFW between treatments. Our results indicated that tissue N, P, K, and Fe levels were not only lower but also deficient in the recycle compared to control treatment. A tissue N concentration below 30 mg⋅g^–1^ is considered as deficient for hydroponically grown lettuce (Campbell, 2000). Optimum range of tissue N, P, and K levels, based on lettuce yield in the field, was 43–56, 4.5–7.5, and 33–64 mg⋅g^–1^, respectively while optimum range of tissue Fe for lettuce was 86–232 mg⋅kg^–1^ (Hartz and Johnstone, 2007). Based on these, tissue N, P, and K levels were lower than optimal in the recycling treatment (Table 3). While Fe was mildly deficient in the control, it was severely deficient in the recycle treatment (Table 3). Although, Cu levels were lower in the control than recycle treatment, the levels were in the sufficiency range (5.6–8.2 mg⋅kg^–1^, Hartz and Johnstone, 2007) in both treatments (Table 3). Tissue Ca and Mg levels were not different between the recycle and control treatments in spite of their high concentration in the tap water (Table 4). Ca uptake is regulated by transpiration rate of plants (Isermann, 1970; Bangerth, 1979). There were no differences in ET between the two solution treatments in experiment 2 (Table 2). This is the likely reason for not observing differences in Ca levels in the plant tissue between the two solution treatments. Further Ca can regulate Mg uptake in plants (Tang and Luan, 2017; Liang and Zhang, 2018), which may have likely resulted in no differences in the levels of Mg in the plant tissue. Therefore, growth reduction in the recycle treatment is mostly due to nutrient deficiencies in the tissue. Plants in the control treatment regularly received freshly prepared nutrient solution with balanced levels of individual nutrients, thereby nutrient deficiencies were not observed in this treatment.

We also observed a significant reduction in SWC, a major component of SFW of lettuce, in the recycle treatment (Table 1). This is likely associated with increased osmotic stress experienced by plants in the recycle treatment. Tap water contained 14 and 38 mg⋅L^–1^ of Na and Cl, respectively (Table 4). It is possible that NaCl accumulated in the recycle solution with time. Accumulation of NaCl can result in osmotic stress effects on plants (Med-Tek Nutrients, 2016; Ding et al., 2018), which likely decreased water uptake of plants in the recycle treatment. This is further supported by an increase in tissue Na levels in the recycle than control treatment (Table 3). High concentration of Na in the tap water (Table 4) likely lead to higher levels of Na in the tissue of plants grown in the recycle treatment. Higher than 5.0 mg⋅g^–1^ of Na in plant tissue can cause injury in plants (WateReuse Foundation, 2007). In spite of being higher than the control treatment, tissue levels of Na were below the injury level in the recycle treatment. Therefore, no visible Na injury symptoms were observed on plants in the recycle treatment. However, an increase in NaCl levels in the solution can potentially reduce water uptake (due to osmotic stress) and subsequently lower SWC and SFW of plants. Collectively, these results indicate that continuously using recycling nutrient solution despite maintaining solution EC at a target level can significantly lower lettuce yield in hydroponic production due to decreased nutrient availability and plant water uptake.

Plant growth differences were observed between the two production systems, when data were pooled from the three solution treatments and four cultivars. The increase in plant growth in the NFT compared to CFT (Supplementary Table 1) is likely due to higher solution temperature in the CFT, especially during the daytime (Supplementary Figure 1). The average air temperature during the study was 23.7°C. The solution temperature increased above air temperature in both the CFT and NFT systems during the daytime, with higher spikes in the CFT (Supplementary Figure 1). It ranged between 0.25 to 1.25 and 0.25 to 0.5°C, respectively in the CFT and NFT systems during different days. The black color of the CFT trays and lids can absorb most of the short wave and infrared radiation from sunlight, which can increase the material temperature. The heat from plastic can be transferred to the solution and increase the temperature of the solution. The optimal temperature for lettuce is between 20 and 24°C (Gent, 2016). Thus, the higher solution temperature in the CFT could have resulted in slower growth compared to the NFT system. However, further research is needed to understand growth differences between the two production systems. Higher SFW and SDW in Black Seeded Simpson than other cultivars is expected as leaf lettuce cultivars can grow at a faster rate than cultivars belonging to other groups (Miller et al., 2020).

### Effect of Accumulation of Unwanted Substances on Solution EC and Nutrient Availability

Evapotranspiration can increase and concentrate nutrients in the solution (Bugbee, 2004), thereby increasing EC of the solution. However, this is not the likely reason for higher EC_adj_ in the recycle than control treatment as there were no differences in ET between the treatments in experiment 2 (Table 2). Alternatively, high levels of Ca, Mg, and HCO_3_ in the tap water (Table 4) can potentially increase EC_adj_. It was reported previously that HCO_3_ have difficulty crossing the lipid bilayer of the root cell membrane (Poschenrieder et al., 2018). When HCO_3_ are added through the tap water, they can accumulate in the solution (Zekki et al., 1996) and increase solution EC. In addition, plants remove Ca and Mg at a slower rate than other elements (Bugbee, 2004; Med-Tek Nutrients, 2016). Consequently, Ca and Mg can accumulate in the nutrient solution and increase solution EC, especially when their levels are high in the tap water. Although we did not measure the concentration of individual ions in the recycle solution, it is likely that higher EC_adj_ in the recycle than control treatment is due to the accumulation of unwanted compounds and/or slowly removed nutrients in the solution because of their high concentration in the tap water. In the control treatment, the concentration of Ca, Mg and HCO_3_ likely was unaffected as old water was discarded regularly. It is important to note that the direct consequence of higher EC_adj_ is a reduction in the volume of concentrated nutrient stock solution added to the recycle solution during EC adjustment. When “apparent” EC of the solution is higher than the target value at the end of each cycle, then a relatively diluted nutrient solution is needed to adjust the EC to the target value. This can reduce the concentration of plant nutrients in the recycling solution compared to control, especially with time. Further, this can reduce the concentration of nutrients available for plant uptake and accumulate in the plant tissue.

### Effects of Discarding Old Solution or Using RO Water on Plant Growth

Our results indicate that recycling solution made from tap water likely accumulated unwanted compounds or slowly consumed nutrients over time, which increased solution EC and reduced the quantity of nutrients added during refill. This further likely reduced tissue nutrient levels and decreased growth. Therefore, discarding old solution after 2 weeks (based on image analysis results) or using RO water with minimal levels of Ca, Mg, and HCO_3_ (based on water quality analyses) should minimize the negative effects of recycling on plants. Supporting this, there were no differences in SFW and SDW observed among the control, 2 WkD, and Rec_RO treatments (Figures 5A,B) regardless of production system and cultivar tested in the experiment. Further, no differences in EC_adj_ between the control and 2 WkD treatments (Figure 5D) suggest that discarding old solution after 2 weeks of use can effectively reduce the accumulation of unwanted and slowly consumed elements in the solution. This further suggests that plants in the 2 WkD treatment likely were not limited with nutrient availability as noted in the conventional recycling treatment. This may have resulted in no growth differences between the control and 2 WkD treatments. The EC_adj_ was lower in Rec_RO than other treatments due to lower target EC (1.1 dS⋅m^–1^) maintained in this treatment. In spite of continuous recycling, no negative effects were observed in the Rec_RO treatment (Figures 5A,B) as insignificant levels of Ca, Mg, and HCO_3_ were measured in the RO water (Table 4). Collectively, these results further support that the growth reduction observed in the continuous recycling treatment is partly due to high levels of Ca, Mg, and HCO_3_ in tap water and their subsequent accumulation in the recycling solution. Root exudates, especially organic acids like benzoic acid, can reduce lettuce growth in closed hydroponic systems (Lee et al., 2006; Hosseinzadeh et al., 2017). While root exudates are important factors affecting lettuce growth in hydroponics, we do not think that their role was significant in our study. The effect of root exudates will likely be present in both tap and RO water based hydroponics. The fact that continuous recycling with RO water did not result in growth reduction suggests that the effect of root exudates may be minimal in our study.

### Practical Remedies to Minimize Negative Effects of Recycling

Our results indicate that preparing recycling solution using RO water or discarding recycling solution after 2 weeks of use can minimize the negative effects of recycling in hydroponic lettuce production. Growers should consider increased operational costs associated with producing RO water, especially in large-scale commercial operations requiring large volume of irrigation water. If discarding is more feasible that using RO water, we recommend that growers discard old solution based on plant growth monitoring as in our study. It is possible to make non-invasive CA measurements using easy-to-use devices like smartphones and publicly available software (Li et al., 2020). Interested growers can visit the our website^1^ to access free software to estimate CA of plants. Growers can track RCGR (as measured in the present study) on a daily basis using CA measurements from image analysis. In our study, RCGR was 0.191 d^–1^ (or 19.1%) and 0.164 d^–1^ (or 16.4%) in the control and recycle treatments, respectively (Table 2 and Figure 4). Similar to our results, Simko et al. (2016) using six lettuce varieties reported RCGR of 0.201 d^–1^ and Li et al. (2020) reported RCGR of 0.21 d^–1^ for lettuce, using image-based assessments under optimal conditions. These reports, combined with our findings, suggest that a RCGR of 19–21% can be expected in lettuce under optimal conditions. Therefore, growers can discard the recycle solution when RCGR between two consecutive days consistently fall below 19–21%.

## Conclusion

Our goals for this study were to evaluate the effects of continuous recycling on solution EC, tissue nutrient concentration and productivity of lettuce, and develop optimal strategies for managing recycling nutrient solution in hydroponic production. The research from this study indicates that continuous recycling with tap water containing moderate to high levels alkalinity can result in apparent increase in solution EC, nutrient deficiencies in the plants, and reduction in shoot growth, in spite of maintaining the solution EC at a target level. In our research, discarding old solution after 2 weeks of recycling effectively mitigated negative effects of recycling on growth. We also provide a solution based on imaging technology for plant growth monitoring to accurately determine the stage when recycling solution made with tap water can be discarded. Alternatively, we found that negative effects of recycling were not observed when RO water was used in production. The information from this study can aid in proper management of recycling nutrient solution in hydroponic production.

## Data Availability Statement

The raw data supporting the conclusions of this article is available upon request.

## Author Contributions

AM contributed to experimental design, experimentation, data collection, and data analyses. RA contributed to manuscript preparation. KN contributed to experimental design, data analyses, and manuscript preparation. All authors contributed to the article and approved the submitted version.

## Conflict of Interest

The authors declare that the research was conducted in the absence of any commercial or financial relationships that could be construed as a potential conflict of interest.

## Footnotes

1 https://www.purdue.edu/hla/sites/cea/la-estimated/
