# Supplementary material for: Recycling Nutrient Solution Can Reduce Growth Due to Nutrient Deficiencies in Hydroponic Production
Source: Front Plant Sci. 2020 Dec 22;11:607643. doi: 10.3389/fpls.2020.607643 (PMC7783079; doi:10.3389/fpls.2020.607643)
Supplement: Supplementary file 1 [file Data_Sheet_1.PDF]

Supplementary Table 1. Effect of production system on shoot fresh weight (SFW), shoot dry weight (SDW), root dry weight (RDW), and root weight ratio (RWR) of lettuce in experiment 3. Data were pooled from the three solution temperature treatments and four cultivars. Means followed by the same letter are not statistically different ($P \leq 0.05$). Standard error of mean is shown in parenthesis.

Production System	SFW $g \cdot plant^{-1}$	SDW $g \cdot plant^{-1}$	RDW $g \cdot plant^{-1}$	RWR
CFT	29.4 (1.5) b	3.0 (0.1) b	2.1 (0.0) b	0.42 (0.008) a
NFT	38.4 (1.7) a	3.4 (0.1) a	2.2 (0.0) a	0.40 (0.008) b

Supplementary Table 2. Effect of cultivar on shoot fresh weight (SFW), shoot dry weight (SDW), root dry weight (RDW), and root weight ratio (RWR) of lettuce in experiment 3. Data were pooled from the three solution temperature treatments and two production systems. Means followed by the same letter are not statistically different ($P \leq 0.05$). Standard error of mean is shown in parenthesis.

Cultivar	SFW $g \cdot plant^{-1}$	SDW $g \cdot plant^{-1}$	RDW $g \cdot plant^{-1}$	RWR
Amd	27.1 (1.8) b	3.0 (0.1) b	2.1 (0.0) a	0.42 (0.010) a
Bss	43.9 (2.9) a	3.8 (0.2) a	2.1 (0.0) a	0.37 (0.011) b
Ced	34.3 (2.0) b	3.1 (0.1) b	2.1 (0.0) a	0.42 (0.009) a
Rex	30.3 (1.9) b	2.9 (0.1) b	2.2 (0.0) a	0.44 (0.012) a

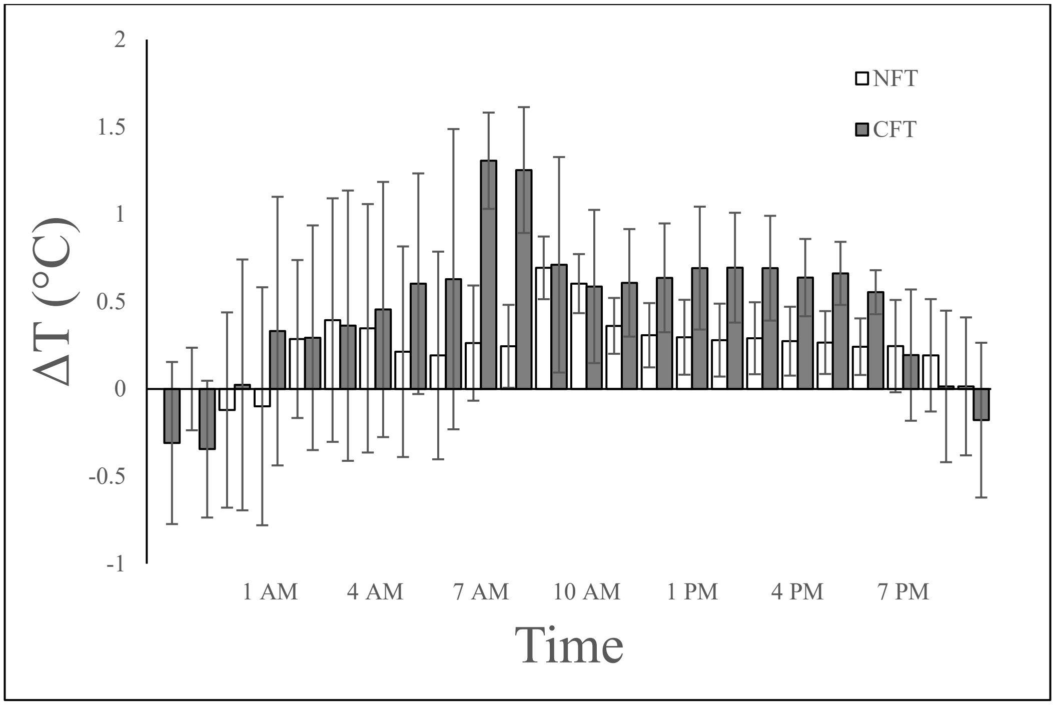

Supplementary Figure 1. Difference between solution temperature and air temperature (ΔT) at different times of the day in the NFT and CFT systems. Data were collected from three replications and seven consecutive days. Error bars represent standard deviation of the mean.
